# Implications of apixaban for dental treatments

**DOI:** 10.4317/jced.53004

**Published:** 2016-12-01

**Authors:** Adrian Curto, Alberto Albaladejo

**Affiliations:** 1 Department of Surgery, Faculty of Medicine, University of Salamanca, Salamanca, Spain

## Abstract

**Background:**

Anticoagulation therapy is used in several conditions to prevent or treat thromboembolism. Recently, new oral anticoagulants have been introduced as alternatives to warfarin and acenocoumarol. In Europe, the European Medicines Agency has approved dabigatran, rivaroxaban and apixaban. Their advantages include: predictable pharmacokinetics, drug interactions and limited food, rapid onset of action and short half-life. However, they lack a specific reversal agent.

**Material and Methods:**

A literature search was conducted through November 2015 for publications in the ISI Web of Knowledge, PubMed, Scopus and Cochrane Library using the keywords “apixaban”, “rivaroxaban”, “dabigatran”, “new oral anticoagulants”, “dental treatment” and “dental implications”. We included studies published in English and Spanish over the last 10 years.

**Results:**

Apixaban has been recently introduced in the daily medical practices for the control of thromboembolism. The number of patients taking apixaban is increasing. Management of patients on anticoagulation therapy requires that dentists can accurately assess the patient prior to dental treatments. It is important for dentists to have a sound understanding of the mechanisms of action and management guidelines for patients taking new oral anticoagulants.

**Conclusions:**

The dentist should consider carefully the management of patients on apixaban. This paper sets out a clinical guidance of dental practitioners treating these patients. There is a need for further clinical studies in order to establish more evidence-based guidelines for dental patients requiring apixaban.

** Key words:**Apixaban, new oral anticoagulants, dental treatment.

## Introduction

In daily dental practice it is common to treat the patients being intervened with oral anticoagulants in order to prevent or treat thromboses. Patients receiving oral anticoagulants have a greater risk of haemorrhage during odontological treatments.

Classically, they were treated exclusively with dicumarinic anticoagulant agents, among which are warfarin and acenocumarol (1), but the disadvantages of these anticoagulants is that their dose must be adjusted specifically for each patient; they interact with many other drugs and with certain foods, and their use requires periodic monitoring using the International Normalized Ratio (INR) (2).

Current research in the field of antithrombotic agents focuses on seeking the ideal oral anticoagulant that will overcome some of the pitfalls of classic oral drugs. Thus, in recent years new anticoagulant agents have appeared, among which are apixaban, riva-roxaban and dabigatran. These new generation drugs do not require periodic laboratory-supervised monitoring and interact less with other drugs and foods (3,4). However, their use in comparison with traditional oral anticoagulants involves economic costs (5,6).

On 20 September 2012 The European Medicines Agency (EMA) authorized the use of apixaban for the prevention of ictus and systemic clots in adult patients with non-valvular atrial fibrillation who had one or more of the following risk factors: ictus or transient ischaemic attack, an age of 75 or above, arterial hypertension, diabetes mellitus and symptomatic cardiac insufficiency equal to or greater than class 2 on the New York Heart Association (NYHA) scale (7). The commercial name of apixaban is Eli-quis®.

Apixaban acts by inhibiting the coagulation factor Xa, indirectly inhibiting the platelet aggregation induced by thrombin. It prevents the formation of thrombin and hence clot formation. In comparison with classic dicumarinic anticoagulants, it inhibits vitamin K-dependent coagulation factors.

The drug is absorbed rapidly and its maximum concentrations are reached at 3-4 hours after administration. Its binding to human plasma proteins is approximately 8% and it has a half-life of 8-15 hours. It is mainly metabolized by cytochrome CYP3A4/5; approximately 25% of the drug is eliminated by the kidney and 27% in urine. The plasma levels of the drug mainly depend on the dose administered and little variation is observed between individuals (8,9).

Currently, there is no antidote to haemorrhages induced by apixaban administration (10). Protamine, vitamin K or plasma transfusions do not affect its anticoagulant effect (11,12).

## Material and Methods

In the present contribution we offer an exhaustive review of the literature found in the ISI Web of Knowledge, PubMed, Scopus and Cochrane Library in November 2015, including articles published in the last 10 years in English and Spanish. The words used were “apixaban”, “rivaroxaban”, “dabigatran”, “new oral anticoagulants”, “dental treatment” and “dental implications” with the “and” boolean operator. Metaanalyses, systematic reviews, clinical trials and case-control studies were considered. Specialized textbooks were also consulted.

## Results

Laboratory tests are available that allow the plasma levels of apixaban to be determined but they are not in wide use and are expensive. Currently, there is no scientific evidence to be able to establish a relationship between the duration of laboratory tests and the likelihood of the occurrence of haemorrhages in patients undergoing treatment with apixaban (13). Among the tests whose results may be altered by apixaban are chromogenic assays to assess anti-factor Xa activity, the prothrombin time and the activated partial thromboplastin time.

-Chromogenic assays to assess anti-factor Xa activity: Anti-factor Xa activity is directly related to the plasma concentration of apixaban. Maximum values are reached with the maximum plasma concentrations of the anticoagulant (14,15).

-Prothrombin time: An increase is seen in the prothrombin time, depending on the plasma concentration in the patients taking apixaban. This increase in the prothrombin time has minimal effects (16).

-Activated partial thromboplastin time: The increase in the activated partial thromboplastin time is insignificant. Like the prothrombin time, this time is not suitable for measuring the effect of apixaban (17-20).

Certain dental treatments that involve a risk of haemorrhage may require temporal interruption of apixaban administration. It is thus of great importance to plan invasive dental treatments entailing a risk of haemorrhage ahead. When dealing with a patient receiving oral anticoagulants, it is necessary to judge the risk of haemorrhage in the dental procedure and the risk of thrombosis arising from the patient’s underlying pathology.

The risk of thrombosis in a patient is gauged by the attendant physician and there are several factors that may govern such risk.

The suspension or not of apixaban and the actual moment of withdrawal depend on the risk of haemorrhage involved in the intervention. It is crucial to know the renal status of the patient to evaluate the moment when the drug is to be suspended (21). The specialist physician will make the decision about whether the drug should be withdrawn or not (22).

In comparison with the new oral anticoagulants, traditional drugs have aroused considerable controversy regarding suspension of the anticoagulant prior to dental procedures (23).

Depending on the risk of haemorrhage of the dental procedure it is possible to classify dental treatments in two groups: procedures with a low risk of haemorrhage and procedures with medium and high risks of haemorrhage.

-Invasive procedures with a low risk of haemorrhage: Considering the low risk of haemorrhage and the half-life of the drug it is not necessary to withdraw treatment with apixaban, with the exception of the dose scheduled for the day on which the dental treatment is to be carried out. The procedure should be performed 12 hours after the last administration of apixaban or at 24 hours if a dose is missed. Low-risk procedures include simple tooth extractions, oral surgery lasting less than 45 minutes and mucogingival surgical procedures (if the haemorrhage is not very extensive). Before performing an invasive procedure, if there has been a previous process of inflammation the risk of haemorrhage will be greater.

Most dental interventions are considered invasive but with a low risk of haemorrhage, such that the suppression of apixaban prior to dental treatment is not necessary. However, to date there are no scientific studies that support such an assertion owing to the short time that this anticoagulant has been available (24).

-Invasive procedures with a medium and high risk of haemorrhage: Within this group, treatment with apixaban must always be withdrawn. The drug should be suspended at least 24 hours previously in the case of medium risk and at least 48 hours in cases of high risk. Owing to the short withdrawal time, no replacement therapy with low molecular weight heparin is necessary. If the period of drug suspension is prolonged, the administration of low-molecular weight heparin is recommended (24-26).

In invasive dental procedures with a low risk of haemorrhage and a medium or high risk if the patient has an altered kidney function, the time of apixaban suspension should be extended ( Table 1). The extraction of more than three teeth at once is considered a medium-high risk procedure, as is the case of oral surgical procedures lasting more than 45 minutes (23-27). In this group, the specialist physician must counsel the withdrawal of apixaban administration.

**Table 1:**
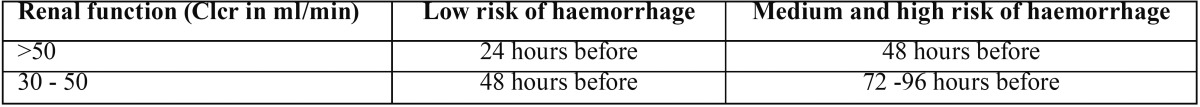
Guide to the suppression of apixaban.

Both in procedures with a low risk of haemorrhage and in those with a medium or high risk, local haemostatic measures must be taken (28-33).

Once the dental intervention has been completed, the time when apixaban treatment should be reinstated is determined by the risk of haemorrhage involved in the invasive procedure performed ( Table 2) (30-33).

**Table 2:**
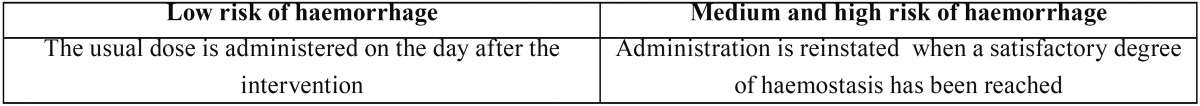
Guide to the reinstatement of treatment with apixaban.

## Conclusions

Patients undergoing treatment with oral anticoagulants have a greater risk of haemorrhaging when receiving invasive dental treatments.

In view of the drawbacks of the classic dicumarinic anticoagulants detected in recent years, new oral anticoagulants have been developed, among which is apixaban. The advantages of these new anticoagulants are as follows: they have predictable pharmacokinetics, they exhibit fewer interactions with drugs and foods, no periodic analytical controls are required and their half-life is shorter. The main drawback is the absence of an efficient antidote if haemorrhages appear.

Clinical experience with the new anticoagulants in dentistry is very limited, but it is to be expected that treatment with these new oral drugs will increase in the future. As health professionals we must be aware of the existence of these new oral anticoagulants and must be ready to report the possible appearance of secondary effects to the pertinent authorities. Finally, protocols must be developed based on the scientific evidence in order to know how these patients should be handled from the point of view of dental interventions.

## References

[1] Mingarro-de-León A, Chaveli-López B (2013). Alternative to oral dicoumarin anticoagulants: Considerations in dental care. J Clin Exp Dent.

[2] Bashir MA, Ray R, Sarda P, Li S, Corbett S (2015). Determination of a safe INR for joint injections in patients taking warfarin. Ann R Coll Surg Engl.

[3] Rillig A, Lin T, Plesman J, Heeger CH, Lemes C, Metzner A (2015). Apixaban, Rivaroxaban and Dabigatran in Patients undergoing Atrial Fibrillation Ablation. J Cardiovasc Electrophysiol.

[4] Imberti D, Gallerani M, Manfredini R (2012). Therapeutic potential of apixaban in the prevention of venous thromboembolism in patients undergoing total knee replacement surgery. J Thromb Thrombolysis.

[5] Barón Esquivias G, Escolar Albaladejo G, Zamorano JL, Betegón Nicolás L, Canal Fontcuberta C, de Salas-Cansado M (2015). Cost-effectiveness Analysis Comparing Apixaban and Acenocoumarol in the Prevention of Stroke in Patients With Nonvalvular Atrial Fibrillation in Spain. Rev Esp Cardiol (Engl Ed).

[6] Lanitis T, Cotté FE, Gaudin AF, Kachaner I, Kongnakorn T, Durand-Zaleski I (2014). Stroke prevention in patients with atrial fibrillation in France: comparative cost-effectiveness of new oral anticoagulants (apixaban, dabigatran, and rivaroxaban), warfarin, and aspirin. J Med Econ.

[7] Budovich A, Zargarova O, Nogid A (2013). Role of Apixaban (Eliquis) in the Treatment And Prevention of Thromboembolic Disease. Pharm Ther.

[8] Frost C, Nepal S, Wang J, Schuster A, Byon W, Boyd RA (2013). Safety, pharmacokinetics and pharmacodynamics of multiple oral doses of apixaban, a factor Xa inhibitor, in healthy subjects. Br J Clin Pharmacol.

[9] Frost C, Wang J, Nepal S, Schuster A, Barrett YC, Mosqueda-Garcia R (2013). Apixaban, an oral, direct factor Xa inhibitor: single dose safety, pharmacokinetics, pharmacodynamics and food effect in healthy subjects. Br J Clin Pharmacol.

[10] Steiner T, Böhm M, Dichgans M, Diener HC, Ell C, Endres M (2013). Recommendations for the emergency management of complications associated with the new direct oral anticoagulants (DOACs), apixaban, dabigatran and rivaroxaban. Clin Res Cardiol.

[11] Connors JM (2015). Antidote for Factor Xa Anticoagulants. N Engl J Med.

[12] Escolar G, Fernandez-Gallego V, Arellano-Rodrigo E, Roquer J, Reverter JC, Sanz VV (2013). Reversal of Apixaban Induced Alterations in Hemostasis by Different Coagulation Factor Concentrates: Significance of Studies In Vitro with Circulating Human Blood. PloS one.

[13] Kanemoto M, Ikeda Y, Fujii T (2015). How to Avoid Adverse Events During Apixaban Therapy in Patients With Atrial Fibrillation. Circ J.

[14] Osanai H, Ajioka M, Masutomi T, Kuwayama T, Ishihama S, Sakamato Y (2015). Measurement of Anti-Factor Xa Activity in Patients on Apixaban for Non-Valvular Atrial Fibrillation. Circ J.

[15] Becker RC, Yang H, Barrett Y, Mohan P, Wang J, Wallentin L (2011). Chromogenic laboratory assays to measure the factor Xa-inhibiting properties of apixaban--an oral, direct and selective factor Xa inhibitor. J Thromb Thrombolysis.

[16] Kanemoto M, Kuhara H, Ueda T, Shinohara T, Oda T, Nakao F (2014). Association of apixaban therapy and prothrombin time in patients with atrial fibrillation. Circ J.

[17] Cheung YW, Barco S, Hutten BA, Meijers JC, Middeldorp S, Coppens M (2015). In vivo increase in thrombin generation by four-factor prothrombin complex concentrate in apixaban-treated healthy volunteers. J Thromb Haemost.

[18] Dale BJ, Chan NC, Eikelboom JW (2016). Laboratory measurement of the direct oral anticoagulants. Br J Haematol.

[19] Cuker A, Husseinzadeh H (2015). Laboratory measurement of the anticoagulant activity of edoxaban: a systematic review. J Thromb Thrombolysis.

[20] Dale BJ, Ginsberg JS, Johnston M, Hirsh J, Weitz JI, Eikelboom JW (2014). Comparison of the effects of apixaban and rivaroxaban on prothrombin and activated partial thromboplastin times using various reagents. J Thromb Haemost.

[21] van Ryn J, Stangier J, Haertter S, Liesenfeld KH, Wienen W, Feuring M (2010). Dabigatran etexilate--a novel, reversible, oral direct thrombin inhibitor: interpretation of coagulation assays and reversal of anticoagulant activity. Thromb Haemost.

[22] Martínez-López F, Oñate-Sánchez R, Arrieta-Blanco JJ, Oñate-Cabrerizo D, Cabrerizo-Merino MD (2013). Clinical diseases with thrombotic risk and their pharmacologycal treatment: how they change the therapeutic attitude in dental treatments. Med Oral Patol Oral Cir Bucal.

[23] Spyropoulos AC, Douketis JD (2012). How I treat anticoagulated patients undergoing an elective pro-cedure or surgery. Blood.

[24] Firriolo FJ, Hupp WS (2012). Beyond warfarin: the new generation of oral anticoagulants and their implications for the management of dental patients. Oral Surg Oral Med Oral Pathol Oral Radiol.

[25] Schulman S, Crowther MA (2012). How I treat anticoagulants in 2012: new and old anticoagulants, and when and how to switch. Blood.

[26] Little JW (2012). New oral anticoagulants: will they replace warfarin?. Oral Surg Oral Med Oral Pathol Oral Radiol.

[27] Cardona-Tortajada F, Sainz-Gómez E, Figuerido-Garmendia J, de Robles-Adsuar AL, Morte-Casabó A, Giner-Muñoz F (2009). Dental extractions in patients on antiplatelet therapy. A study conducted by the Oral Health Department of the Navarre Health Service (Spain). Med Oral Patol Oral Cir Bucal.

[28] Faraoni D, Samama CM, Ranucci M, Dietrich W, Levy JH (2014). Perioperative management of patients receiving new oral anticoagulants: an international survey. Clin Lab Med.

[29] Hankey GJ, Norrving B, Hacke W, Steiner T (2014). Management of acute stroke in patients taking novel oral anticoagulants. Int J Stroke.

[30] Lai A, Davidson N, Galloway SW, Thachil J (2014). Perioperative management of patients on new oral anticoagulants. Br J Surg.

[31] Dincq AS, Lessire S, Douxfils J, Dogné JM, Gourdin M, Mullier F (2014). Management of non-vitamin K antagonist oral anticoagulants in the perioperative setting. Biomed Res Int.

[32] Baron TH, Kamath PS, McBane RD (2013). Management of antithrombotic therapy in patients undergoing invasive procedures. N Engl J Med.

[33] Sié P, Samama CM, Godier A, Rosencher N, Steib A, Llau JV (2011). Surgery and invasive procedures in patients on long-term treatment with direct oral anticoagulants: thrombin or factor-Xa inhibitors. Recommendations of the Working Group on Perioperative Haemostasis and the French Study Group on Thrombosis and Haemostasis. Arch Cardiovasc Dis.

